# Experimental Comparison between 4D Stereophotogrammetry and Inertial Measurement Unit Systems for Gait Spatiotemporal Parameters and Joint Kinematics

**DOI:** 10.3390/s24144669

**Published:** 2024-07-18

**Authors:** Sara Meletani, Sofia Scataglini, Marco Mandolini, Lorenzo Scalise, Steven Truijen

**Affiliations:** 1 Department of Industrial Engineering and Mathematical Sciences, Università Politecnica delle Marche, via Brecce Bianche 12, 60131 Ancona, Italy; s1107227@studenti.univpm.it (S.M.); m.mandolini@staff.univpm.it (M.M.); l.scalise@staff.univpm.it (L.S.); 2 4D4ALL Lab, Department of Rehabilitation Sciences and Physiotherapy, Center for Health and Technology (CHaT), Faculty of Medicine and Health Sciences, MOVANT, University of Antwerp, 2000 Antwerpen, Belgium; steven.truijen@uantwerpen.be

**Keywords:** gait spatiotemporal parameters, 4D scanning, wearable mocap system

## Abstract

(1) Background: Traditional gait assessment methods have limitations like time-consuming procedures, the requirement of skilled personnel, soft tissue artifacts, and high costs. Various 3D time scanning techniques are emerging to overcome these issues. This study compares a 3D temporal scanning system (Move4D) with an inertial motion capture system (Xsens) to evaluate their reliability and accuracy in assessing gait spatiotemporal parameters and joint kinematics. (2) Methods: This study included 13 healthy people and one hemiplegic patient, and it examined stance time, swing time, cycle time, and stride length. Statistical analysis included paired samples *t*-test, Bland–Altman plot, and the intraclass correlation coefficient (ICC). (3) Results: A high degree of agreement and no significant difference (*p* > 0.05) between the two measurement systems have been found for stance time, swing time, and cycle time. Evaluation of stride length shows a significant difference (*p* < 0.05) between Xsens and Move4D. The highest root-mean-square error (RMSE) was found in hip flexion/extension (RMSE = 10.99°); (4) Conclusions: The present work demonstrated that the system Move4D can estimate gait spatiotemporal parameters (gait phases duration and cycle time) and joint angles with reliability and accuracy comparable to Xsens. This study allows further innovative research using 4D (3D over time) scanning for quantitative gait assessment in clinical practice.

## 1. Introduction

Gait analysis applies anatomical and biomechanical principles to systematically understand and characterize human locomotion [1]. The act of gait involves the complex interaction of muscle forces on bones, rotations through multiple joints, and physical forces that act on the body. Walking also requires motor control and motor coordination; thus, it represents a complex neuro-muscular activity [2]. Applying gait analysis techniques allows clinicians to obtain kinematic data, such as the angles between various body segments, dynamic data, which includes moments and forces developed during movement, and electromyographic data, providing information on muscle activation and deactivation. The gait analysis study is conducted either for clinical purposes or research. It represents a powerful tool that enables early diagnosis of diseases and their complications and helps to find the best treatment [3,4]. Many different medical conditions, such as multiple sclerosis, Parkinsonism, and stroke, can interfere with walking [1]. Most of them are neurodegenerative diseases which limit everyday functional abilities and have become more prevalent in recent decades [4,5]. Traditionally, gait analysis in clinical settings has relied on subjective methods, mainly through observation. As a result, the evaluator’s experience determines the assessment’s findings and the course of treatment. Systems that enable more accurate and repeatable gait analysis are required, because this evaluation may exhibit notable inter and intra-observer variability and gait impairment characteristics that are invisible to the human eye [6]. 

Indeed, quantitative assessments of mobility performance offer significant advantages from various perspectives, including social, clinical, and patient-centered dimensions. These measurements play a crucial role in giving clinicians essential insights into health status and cognition, thereby providing valuable information about the severity and progression of diseases. They also contribute to enhancing patients’ overall quality of life: for example, these assessments support the fall risk evaluation, allowing for preventive measures to reduce the likelihood of falls. Another advantage is reducing the significant burden placed on relatives and caregivers and decreasing socio-economic costs associated with healthcare [7]. 

In gait analysis, various measurement systems capture and assess different aspects of human walking. A marker-based motion capture system is the gold standard technique for obtaining three-dimensional kinematic measurements of joints [8]. This kind of system employs reflective markers strategically placed on anatomical landmarks, according to the recommendations set by the International Society of Biomechanics (ISB) [9]. The markers’ trajectories are simultaneously recorded by multiple cameras or optoelectronic sensors, providing a comprehensive assessment of the motion of a specific body segment within a three-dimensional space.

Despite its recognized accuracy, marker-based systems have certain limitations. Firstly, the process involves the attachment of numerous markers to the body, making it a time-consuming task that needs to be repeated for each new subject. Secondly, skilled personnel must correctly apply these markers, introducing an operator-dependent aspect to the system. Thirdly, the markers themselves may impact the natural fluidity of motion and are susceptible to soft tissue artifacts (variations in movement between the markers on the skin surface and the underlying bone), leading to potential inaccuracies in measurements. Lastly, the marker-based system tends to incur higher costs than markerless alternatives. While marker-based motion capture systems offer unparalleled accuracy, these drawbacks underscore the need for ongoing advancements and considerations when selecting the most suitable method for specific research or clinical applications [10].

The need to overcome all these drawbacks has led to the research and development of markerless, inexpensive, small, lightweight, and portable devices named Inertial Sensors (IS). These devices can evaluate movements by gauging the inertia of a suspended mass. Due to significant advancements in Micro-Electro-Mechanical Systems (MEMS), it is now possible to fabricate millimeters-sized devices that guarantee precise measurements. Such devices typically feature an integrated circuit (IC) interface, incorporating an analog signal-conditioning block for amplifying and filtering the recorded signal and an analog-to-digital interface for converting the acquired data into a digital format. Various measuring principles, including piezoelectric and piezoresistive methods, are available. However, some accelerometers in the market utilize capacitive measurements to transform displacement into an electric signal. This type of accelerometer has minimal power requirements, making it particularly well-suited for developing wearable, wireless, and battery-powered devices. Acceleration sensors have the unique capability to measure acceleration along a designated direction. These transducers are categorized based on the number of sensitive directions, such as one-axis, two-axis, or three-axis accelerometers. In scenarios necessitating information about the device’s rotation, such as in gait analysis, angular velocity sensors (gyroscopes) must be incorporated. When a three-axis gyroscope is combined with a three-axis accelerometer, the assembly is called an Inertial Measurement Unit (IMU) [11,12,13,14].

IMUs have a lot of issues. IMUs measure acceleration and angular velocity and then integrate these measurements over time to obtain position and orientation [11,12,13,14]. However, this integration process can lead to cumulative errors over time: this phenomenon is known as integration drift [11]. This issue can cause a gradual deviation from the actual position, especially in long-duration captures.

Additionally, IMU systems could be sensitive to magnetic disturbances caused by near ferromagnetic devices [11,12,13,14]. This limitation has significant effects on the signal acquired, meaning it is usually not enough, even if algorithms that compensate for those disturbances are used. Furthermore, even if IMUs are reliable for assessing gait analysis parameters, their accuracy and precision are lower than optoelectronic systems, especially for estimating stride length [11,12,13,14]. In addition, IMUs require positioning the sensors with Velcro straps on the human body or integrating them into clothing. Such requirements are time-consuming and affect the natural and unrestricted movement during walking. In contrast, using the emerging three-dimensional (3D) temporal scanning (also known as 4D scanning) method for gait analysis assessment can solve the previous issue. 

It is possible to dynamically capture the full body shape of a subject during a motor task (e.g., gait) with 4D scanning (3D + time). The 3D temporal scanning technique has been primarily used for applications such as anthropometry, skin-surface area, body size and shape, and 3D visualization for applications such as apparel (e.g., smart clothing [15,16], implants, orthotics, and prosthetics [17]). The 3D time scanning or 4D scanning [18] method in gait analysis [19] would have significant advantages over traditional motion capture methods. For example, they do not need markers or IMU attachment, which could lead to artifacts and cause a non-free natural movement. In addition, the possibility of correlating the full-body shape variation with body mass composition during a task such as gait analysis is essential in pathologies such as obesity [20]. Ruescas Nicolau et al. [19] proposed a comparative study between marker-based stereophotogrammetry and 3D temporal scans to assess joint kinematics. However, their work did not include spatiotemporal gait parameters such as stance time, swing time, cycle time, and stride length. Consequently, it is necessary to overcome this issue by exploring gait spatiotemporal parameters using 3D temporal scanning technology.

The present work aims to solve this issue by comparing a 3D temporal scanning system (Move4D) with an inertial motion capture system (Xsens) to evaluate its reliability and accuracy in assessing gait spatiotemporal parameters and joint kinematics.

After this introduction, the paper presents the methodology (Section 2) behind the comparison. It describes the test protocol, the two motion capture tools, the signal post-processing, and the statistical analysis. Section 3 gives the spatiotemporal parameters results to evaluate the inter-trial variability, inter-subject variability, intra-class correlation coefficients, paired *t*-test, and Bland–Altman plots (see Appendix A) for the measurements between Move4D and Xsens. Section 4 discusses the results to draft conclusions (Section 5). 

## 2. Materials and Methods

An experimental comparison between 4D stereophotogrammetry and inertial measurement unit systems for gait spatiotemporal parameters and joint kinematics will be presented on thirteen healthy people and one hemiplegic patient during gait. This section will describe the protocol, instrumentation, related data processing, and statistical analysis. The objective is to demonstrate that Move4D (IBV, Valencia, Spain) can assess gait spatiotemporal parameters and kinematic data with reliability and accuracy comparable to that of Xsens (Movella, NL, USA).

### 2.1. Protocol

Thirteen healthy people (9 females and 4 males) and one hemiplegic patient (female) were recruited for this study. The number of participants was chosen according to Julious [21], who suggested that the number of subjects must be at least twelve for a pilot study. The mean and standard deviation (SD) of the anthropometric characteristics of the subjects are reported in Table 1.

All the participants signed informed consent to participate in the study, which the Antwerp University Hospital (UZA-UA) Ethics Committee approved. The experiment was performed in the 4D4ALL laboratory at RevArte Rehabilitation Hospital. The 4D4ALL laboratory comprises several devices and tools that can be synchronized for precision and personalized medicine measurements during a motor task such as walking.

### 2.2. Instrumentation

**Figure 1:**
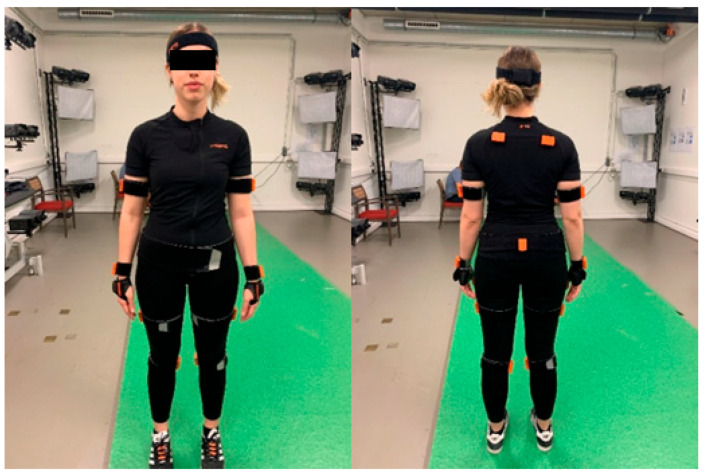
Xsens sensors placement. Front (**left**) and back (**right**) views.

**Figure 2:**
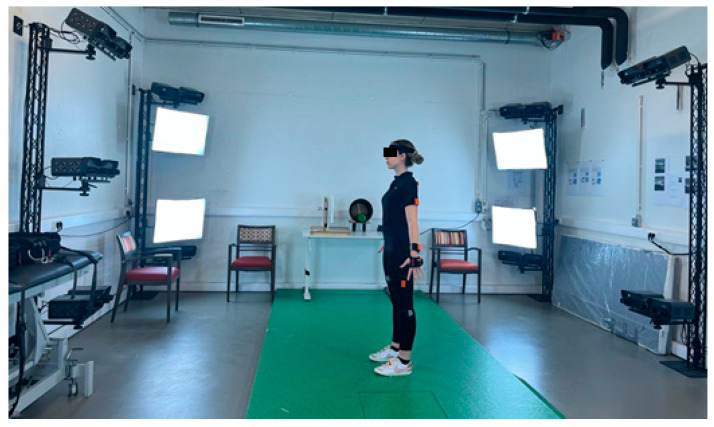
Synchronization of both devices (Xsens) and Move4D in A-Pose.

During this study, two different motion capture tools of the 4D4ALL lab were considered: the Move 4D (IBV, Valencia, Spain) dynamic scanner device and the wearable mocap system Xsens (Movella, Henderson, NL). The Move4D scanning volume is 2 m (width) × 2 m (depth) × 3 m (height). It is composed of 12 modules positioned on columns on a truss structure. It is equipped with built-in lighting systems (Figure 1 and Figure 2). The modules are composed of a combination of infrared and RGB cameras able to capture dynamic (3D over time) full-body shape variation and kinematics at 178 frames per second (fps) with an accuracy of less than 1 mm. At the same time, the second tool is a mocap system composed of 17 inertial wearable measurement units (orange units in the figures) attached partially to the human body by Velcro straps or attached to a smart shirt, gloves, and band as in Figure 1 and Figure 2. Both systems have been synchronized at 60 Hz and linked through a coaxial cable. The cable has been put in the output for Xsens and the input for Move4D. As soon as the capture with Xsens is launched, Xsens sends the trigger to Move4D to start the acquisition.

The experiment’s first phase consisted of capturing in the A-Pose, during which the subject stands upright with legs separated at the shoulder level and with the arms slightly abducted at 45 degrees (4 frames are acquired at 90 fps with high resolution).

The second step of the experiment is the Xsens segment calibration, which makes it possible to align the motion trackers with the body segments of the subject. The procedure consists of standing for some seconds in N-pose (or A-pose or T-pose), then start walking for some seconds and then standing again. The calibration becomes difficult for some subjects (e.g., stroke patients) who cannot maintain the standing position. 

The last step is capturing the gait, where the subjects were asked to naturally walk on the ground for three meters to cover all the calibration volume of Move4D and to capture at least one complete gait cycle. The subjects were not asked to start with the left or right foot to guarantee a natural movement. For each subject, at least three trials have been captured. The sampling frequency of the acquisition is the same for the two measurement systems (60 Hz). The sampling frequency of Xsens for acquiring the full-body motion is 60 Hz, so the sampling frequency of Move4D has been set at 60 Hz, too. For this purpose, in the software Move4D V1.5, a new “Mode” for the synchronized acquisition with Xsens has been created, with 180 frames acquired at 60 fps and high resolution. The data were processed using the MATLAB software environment (version R2023a).

For both Move4D and Xsens, the trajectory of the points has been considered. Move4D allows obtaining the trajectory of all 50,000 anatomical points starting from the mesh of the body contained in the wavefront (OBJ) file [22]. Once set the correspondence between mesh vertex IDs and anatomical points of the kinematic model (in this case, the “Plugin Gate” model) [23], it is possible through a Python script to convert the OBJ file in a Track Row Column (TRC) file [22] which contains the trajectory (3D coordinates) of the chosen points. For Xsens, the trajectory of the points of interest has been taken from the Excel file automatically computed by the MVN Software from the “Segment Position” sheet. For both systems, the 3D coordinates of the points have been filtered with a low pass 4th order Butterworth filter with a cut-off frequency at 8 Hz to remove the noise.

### 2.3. Post-Processing

**Figure 3:**
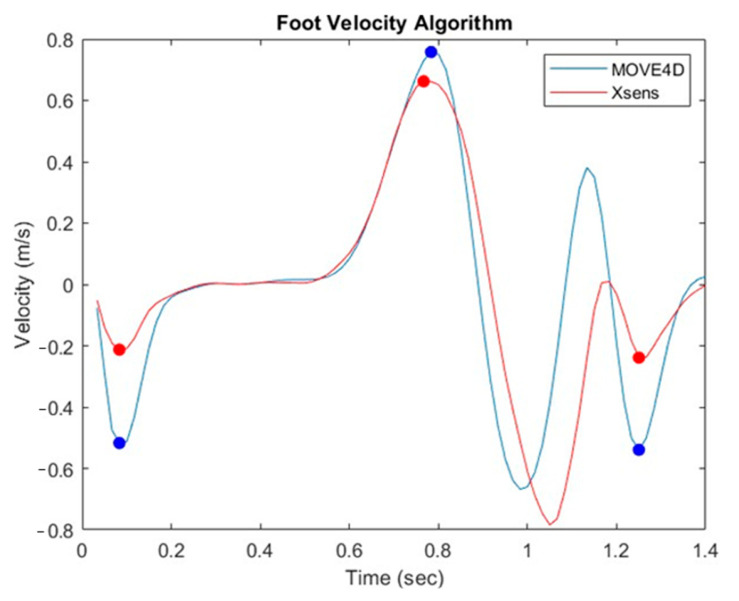
Foot center velocity with identification of step events [24]. The first dot minimum represents the time of the initial heel strike. In contrast, the final dot minimum is the final heel strike, so they mark the start at the end of the gait cycle. The absolute dotted maximum instead represents the toe-off.

To compute the spatiotemporal parameters, finding the heel strike and toe-off time instants of the gait cycle is necessary. To see these time instants, the Foot Velocity Algorithm (FVA) has been applied [24]. This algorithm considers the heel and toe markers’ vertical (longitudinal) coordinates. It applies a low pass 4th order Butterworth filter to remove the noise. A new signal representing the foot center is created by calculating the midpoint of the heel and toe marker locations. The vertical velocity of the foot center is calculated by taking the first derivative of the vertical coordinates using finite difference equations. This vertical velocity of the foot center has a simple characteristic shape repeated for each gait cycle during normal gait, as reported in Figure 3. 

Some changes have been made to adapt the FVA algorithm, validated using the Vicon system, to the data from Move4D and Xsens. First, while the original FVA uses the 2nd metatarsal head as the toe marker, the 3rd metatarsal head has been used in this study. The reason for this is that the output data of Move4D provides the trajectory of the 3rd metatarsal head by default. For the Xsens signal, the trajectory of the foot’s center has been directly used since it is automatically available from the Excel file from MVN software (Movella, NL, USA).

Moreover, the FVA, to detect the initial and final heel strikes, applies a threshold on the height of the heel marker set at 35% of the range of heel heights encountered during the trial. In the present study, instead, a threshold on the velocity has been applied, and in particular, the minima lower than −0.6 m/s have been canceled out. The reason is that after the toe-off, as Figure 3 shows, the velocity of the foot center is characterized by an absolute minimum, representing the foot’s oscillation during the swing phase. This minimum does not represent the final heel strike because the heel marker is still in the swing phase and too far from the ground.

The amplitude of the foot center velocity of Move4D and Xsens show some differences especially in the initial and final heel strike. The reason can be related to the attachment of the Xsens sensor on the foot because it is places over the shoe. Despite that, our focus is on the time instants where hell strike and toe off occur which were similar in the two systems.

The duration of the stance phase has been computed as the difference between the toe-off time instant and the first heel strike time instant. Instead, the duration of the swing phase has been calculated as the difference between the final heel strike time instant and the toe time instant. The total duration of the gait is equal to the sum of the stance and swing phases.

To compute the stance phase and swing phase percentages, the respective time duration was multiplied by 100 and divided by the total duration of the gait. The stride length has been computed as the difference between the sagittal coordinate of the heel marker in the final heel strike and the initial heel strike. The kinematic data and the spatiotemporal parameters have been processed with MATLAB software (version R2023a). The kinematic data were taken from the BioVision Hierarchy (BVH) file [22] for Move4D and from the sheet in the Excel file containing the joint angles for Xsens. The signals have been smoothed using an average moving filter to reduce the noise. For each subject and each joint of the first trial, the range of motion has been computed as the difference between the maximum and the minimum values of the signals. 

### 2.4. Statistical Analysis

IBM SPSS Statistics software (version 29.0.1.0) was used for statistical analysis. The mean and standard deviation between trials for each subject have been computed to evaluate the inter-trial variability for both systems. After the computation of all subjects’ mean and standard deviation, the 95% confidence interval was calculated. To assess the inter-subject variability for both systems, the mean, the standard deviation, and the 95% confidence interval for the mean between measurements were computed for each trial. A paired *t*-test with Move4D and Xsens measurements has been performed for each spatiotemporal parameter. Additionally, Bland–Altman plots have been constructed. This evaluation has been done for each trial and the 13 healthy subjects.

The intraclass correlation coefficient was computed to evaluate the reliability of the measure. This analysis has been done for each parameter, and the two classes are the measurement with Move4D and the measurements with Xsens.

Lastly, for the kinematic analysis, only the first trial was considered. The root-mean-square error (RMSE) between the range of motions captured by Move4D and Xsens has been computed to evaluate the accuracy. Moreover, the mean, the standard deviation, and the 95% confidence interval for the range of motion have been reported for both measurement systems, the healthy subjects, and the hemiplegic patient.

## 3. Results

The results of the spatiotemporal parameters involve the evaluation of the inter-trial variability, the inter-subject variability, the computation of intra-class correlation coefficients, the paired *t*-test, and the Bland–Altman plots (see Appendix A) for the measurements between Move4D and Xsens. 

### 3.1. Inter-Trial Variability

Table 2 shows the mean, the standard deviation, and the 95% confidence interval (CI) of the measurements across the trials for thirteen healthy subjects on ten trials. First, the mean and standard deviation between the trials for each subject were computed. Then, the mean and standard deviations between the subjects were calculated. The trials do not show high variability, as the values of the standard deviations are low for each parameter. The only comment is for the stride length: for Xsens, the standard deviation on the stride length (SD = 0.13 m) is more than two times higher than that of Move4D (SD = 0.05 m). Then, it can be stated that for Xsens, the variance between trials for stride length is higher than for Move4D.

### 3.2. Inter-Subjects Variability

Table 3, Table 4 and Table 5 show the mean, the standard deviation, and the 95% confidence interval for the mean measurements of the 13 healthy subjects for the first, second, and third trials, respectively.

For all three trials, the variability between subjects shows higher results for the measurements obtained with Xsens. The values of the standard deviations for Xsens are equal to or higher than those obtained with Move4D. This trend occurs for all the spatial parameters but mainly for the stride length. For all the trials, the standard deviation obtained for Xsens is more than two times higher than Move4D.

Table 6 shows, both for Move4D and Xsens, the mean, the standard deviation, and the 95% confidence interval of the mean values of the spatiotemporal parameters for the hemiplegic patient among the three trials.

Comparing Table 2 and Table 6 shows that the values of the various means for the hemiplegic patient are outside the 95% CI found for the healthy subjects. In particular, it can be observed that both for Move4D and Xsens, the mean values of the stance time, the stance percentage, and the cycle time for the hemiplegic patient are higher (or at least equal) to the upper limit of 95% CI of the mean of the healthy subjects. For the swing phase and the stride length, the values for the hemiplegic patient are lower than the lower limit of the 95% CI of the mean for the healthy subjects. This result is valid for all the trials; thus, the Move4D system can distinguish pathological subjects from healthy ones.

### 3.3. Paired Samples t-Test and Bland–Altman Analysis

Table 7 shows the results of a paired samples *t*-test between the values of the spatiotemporal parameters measured with Move4D and Xsens for the first trial. 

Table 7 shows no significant difference between the measurement systems for stance time, swing time, and stance percentage, as the mean differences are low (*p* > 0.05). For the cycle time and the stride length, a significant difference between Move4D and Xsens has been found (*p* < 0.05). In particular, the mean difference is 0.272 m for the stride length. 

**Figure A1:**
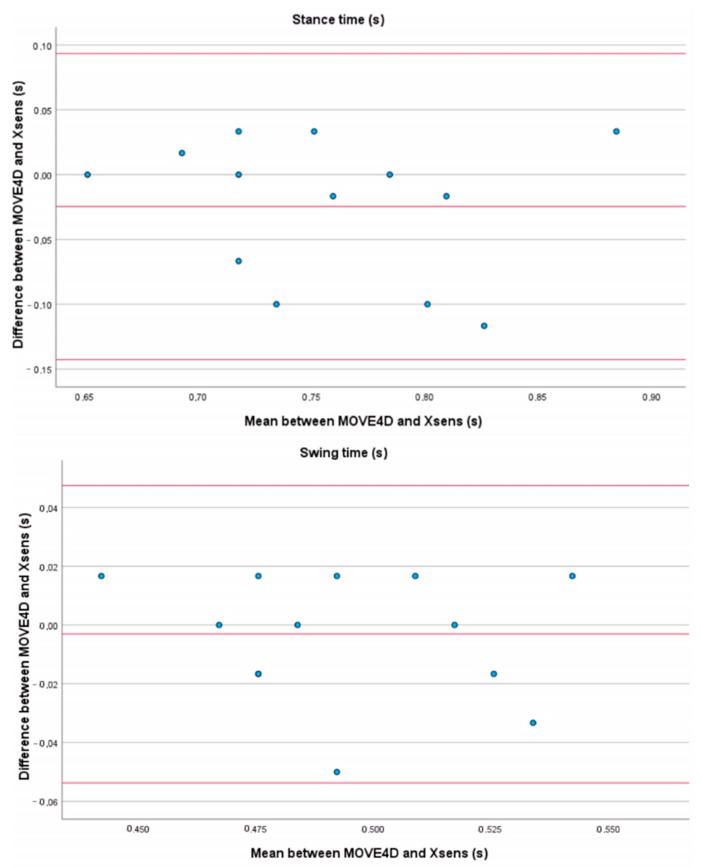
Bland–Altman plots of stance and swing duration differences between measurements analyzed by Move4D and Xsens for the first trial. The central red line represents the mean difference. In contrast, the upper and lower red lines represent the upper and lower limits of the 95% CI, respectively. For stance time, the mean difference is −0.025 s, while for swing time, it is −0.003 s.

**Figure A2:**
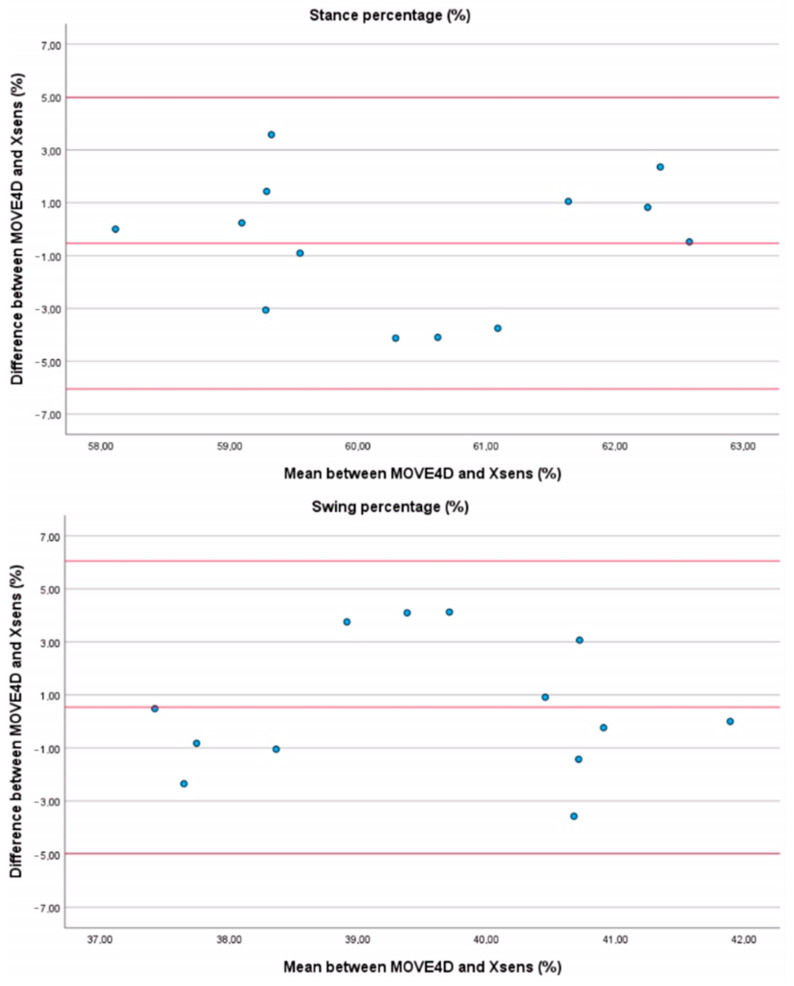
Bland–Altman plots of stance and swing percentage differences between measurements analyzed by Move4D and Xsens for the first trial. The central red line represents the mean difference. In contrast, the upper and lower red lines represent the upper and lower limits of the 95% CI, respectively. The mean difference for the stance percentage is −0.535%, while the swing percentage is 0.535%.

**Figure A3:**
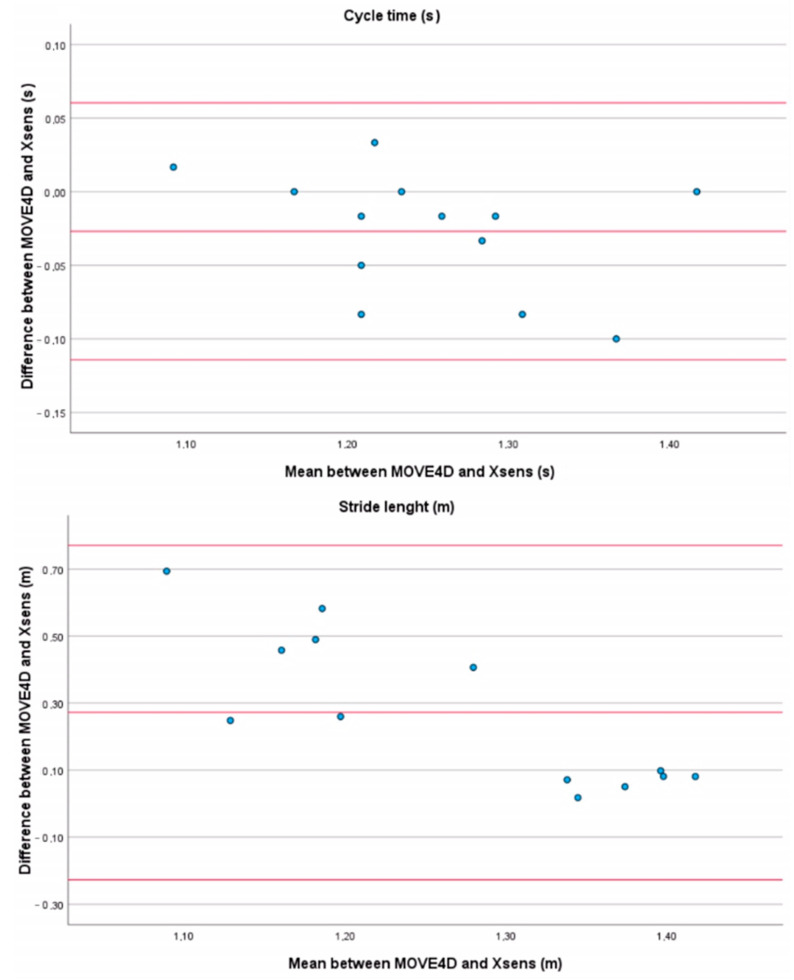
Bland–Altman plots cycle time and stride length differences between measurements analyzed by Move4D and Xsens for the first trial. The central red line represents the mean difference. In contrast, the upper and lower red lines represent the upper and lower limits of the 95% CI, respectively. For cycle time, the mean difference is −0.027 s, while for stride length, it is 0.272 m.

The dispersion of the differences between Move4D and Xsens for each parameter of the first trial is shown in the Bland–Altman plots (Figure A1, Figure A2 and Figure A3) in Appendix A. As can be observed, for the stance time, swing time, stance percentage, swing percentage, and cycle time, the mean difference between Move4D and Xsens is around zero, and the graphs do not show a trend. The mean difference’s value is far from zero for the stride length. As the mean between Move4D and Xsens increases, the difference decreases. For all the parameters, there are no outliers (values outside the 95% CI).

Table 8 shows the results of a paired samples *t*-test between the values of the spatiotemporal parameters measured with Move4D and Xsens for the second trial. In this case, the stride length is the only parameter that shows a significant difference between the estimation with Move4D and Xsens (*p* < 0.05). The difference is insignificant for all of the other parameters (*p* > 0.05), as the low mean difference values demonstrate.

**Figure A4:**
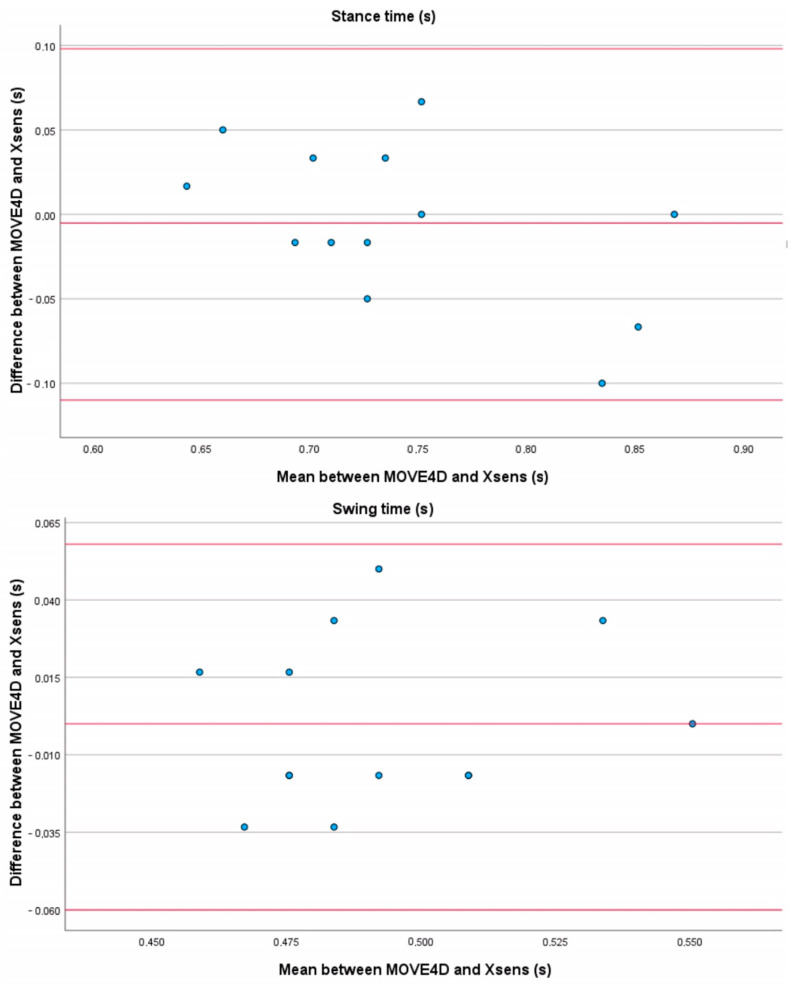
Bland–Altman plots of stance and swing duration differences between measurements analyzed by Move4D and Xsens for the second trial. The central red line represents the mean difference. In contrast, the upper and lower red lines represent the upper and lower limits of the 95% CI, respectively. For stance time, the mean difference is −0.006 s, while the mean of swing time is −0.001 s.

**Figure A5:**
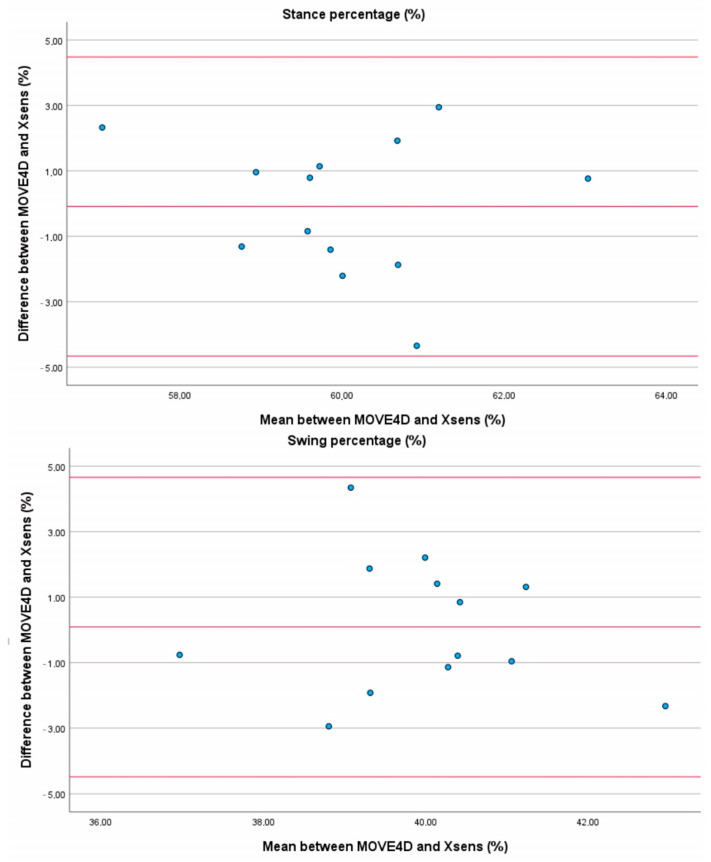
Bland–Altman plots of stance and swing percentage differences between measurements analyzed by Move4D and Xsens for the second trial. The central red line represents the mean difference. In contrast, the upper and lower red lines represent the upper and lower limits of the 95% CI, respectively. For the stance percentage, the mean difference is −0.089%, while for the swing percentage is 0.089%.

**Figure A6:**
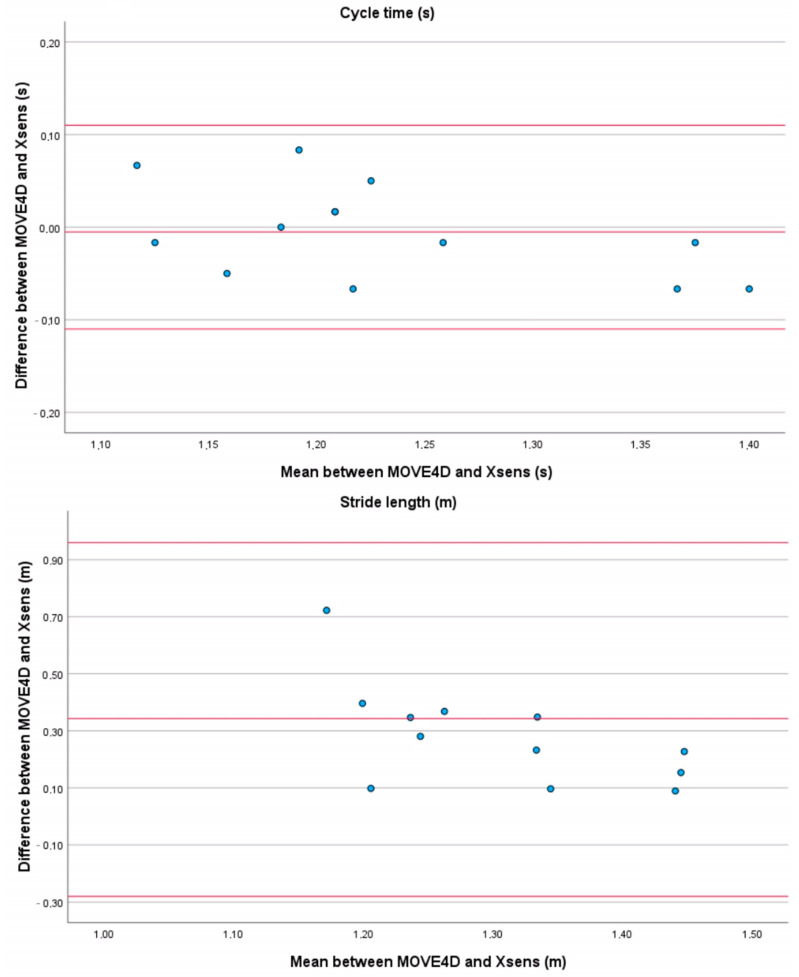
Bland–Altman plots cycle time and stride length differences between measurements analyzed by Move4D and Xsens for the second trial. The central red line represents the mean difference. In contrast, the upper and lower red lines represent the upper and lower limits of the 95% CI, respectively. For cycle time, the mean difference is −0.006 s, while for stride length, it is 0.344 m.

Figure A4, Figure A5 and Figure A6 show the Bland–Altman plots of the spatiotemporal parameter differences between measurements obtained with Move4D and Xsens for the second trial. The same considerations as trial one can be applied to the Bland–Altman plots.

Table 9 shows the results of a paired samples *t*-test between the values of the spatiotemporal parameters measured with Move4D and Xsens for the third trial. In this case, the same comments can be made for Table 8. 

**Figure A7:**
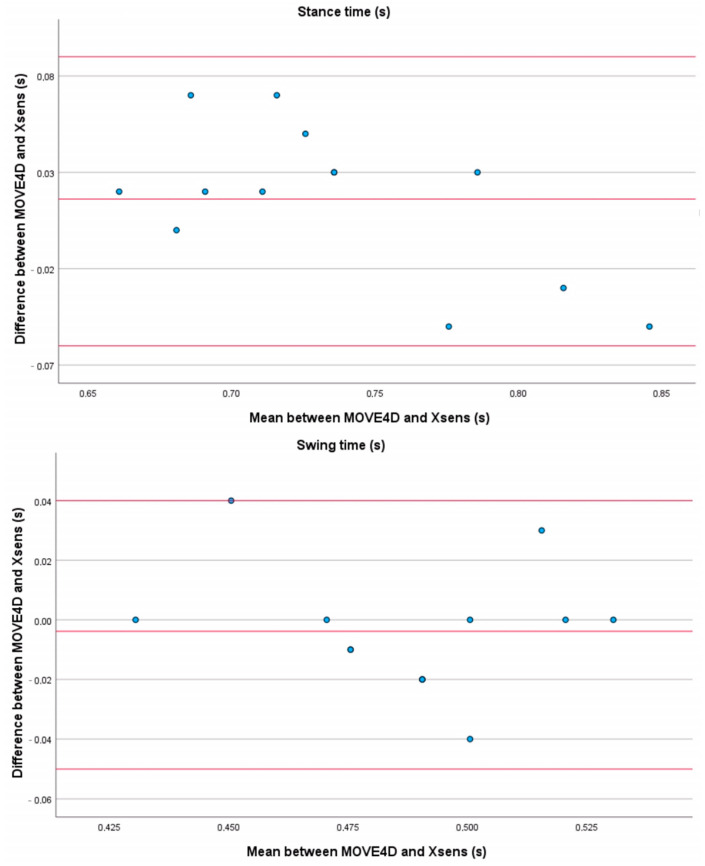
Bland–Altman plots of stance and swing duration differences between measurements analyzed by Move4D and Xsens for the third trial. The central red line represents the mean difference. In contrast, the upper and lower red lines represent the upper and lower limits of the 95% CI, respectively. For stance time, the mean difference is 0.016 s, while for swing time, it is −0.004 s.

**Figure A8:**
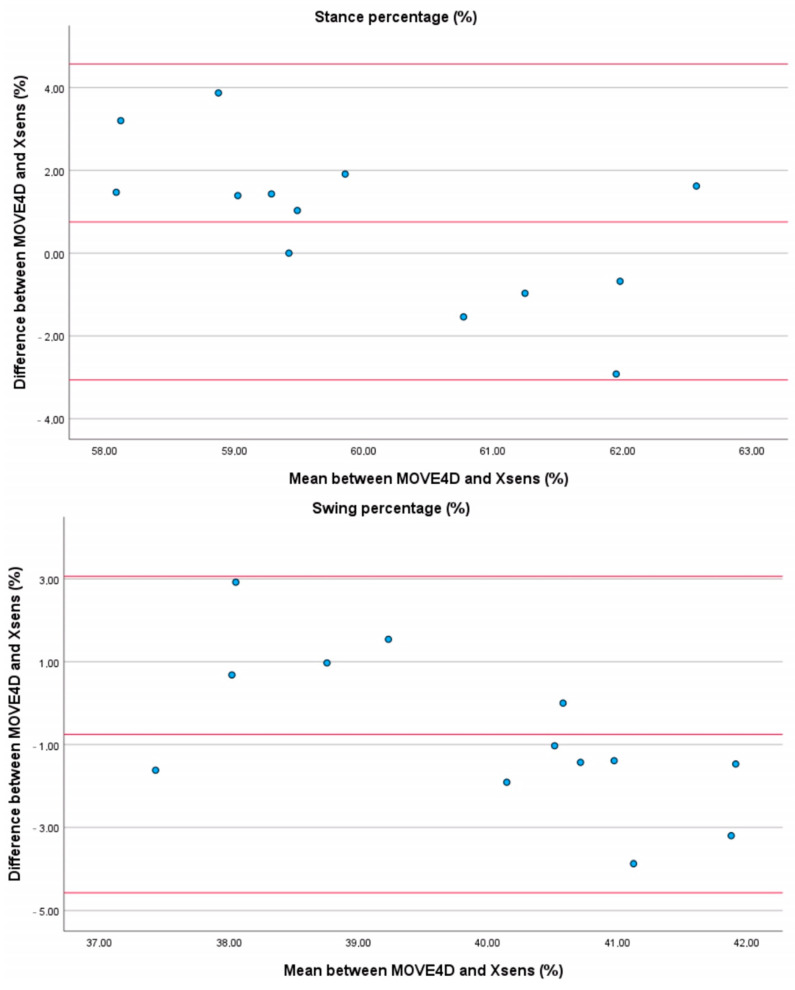
Bland–Altman plots of stance and swing percentage differences between measurements analyzed by Move4D and Xsens for the third trial. The central red line represents the mean difference. In contrast, the upper and lower red lines represent the upper and lower limits of the 95% CI, respectively. For the stance percentage, the mean difference is 0.755%, while for the swing percentage is −0.755%.

**Figure A9:**
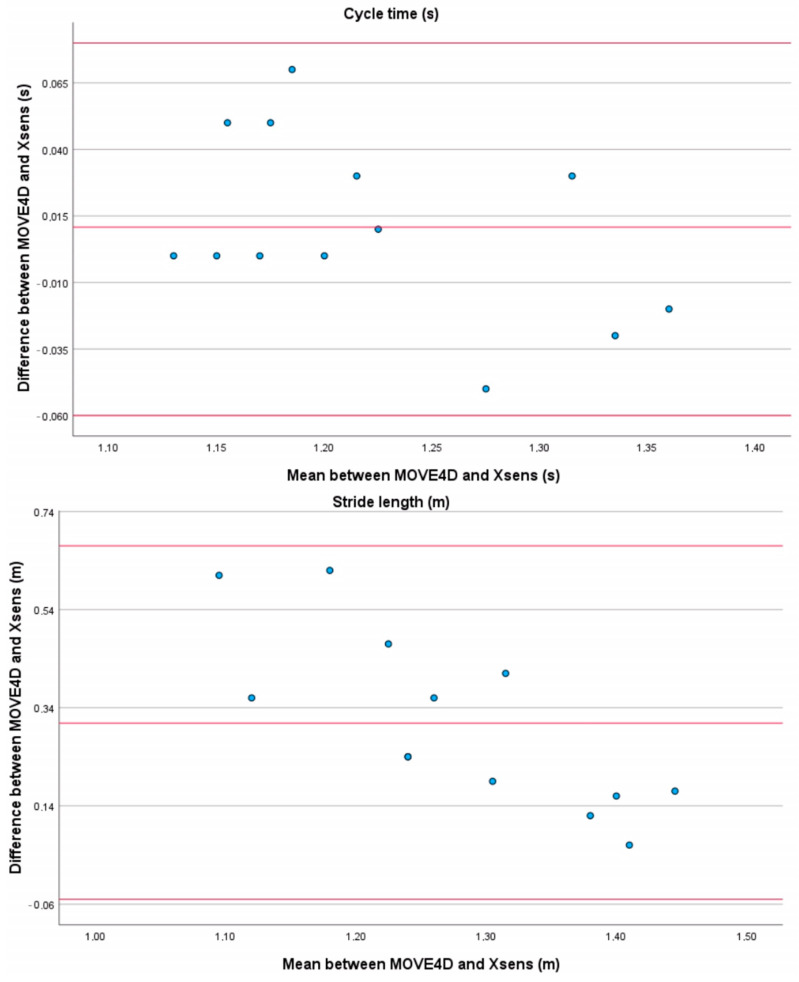
Bland–Altman plots cycle time and stride length differences between measurements analyzed by Move4D and Xsens for the third trial. The central red line represents the mean difference. In contrast, the upper and lower red lines represent the upper and lower limits of the 95% CI, respectively. For cycle time, the mean difference is 0.011 s, while for stride length, it is 0.308 m.

The Bland–Altman plots for the third trial are shown in Figure A7, Figure A8 and Figure A9. The same considerations can be made in trials one and two for the Bland–Altman plots. 

### 3.4. Intra-Class Correlation Coefficient

The intra-class correlation coefficient (ICC) has been used to evaluate the degree of agreement between the measurements obtained with Move4D and Xsens. It was computed for each trial, considering the thirteen healthy subjects.

Table 10 shows the ICC for each parameter measured with Move4D and Xsens for the first trial. Two-way mixed effects, absolute agreement, and single-rater ICC have been computed for all the parameters except cycle time and stride length. In these two cases, two-way mixed effects, consistency, and single rater ICC have been evaluated since the previous results on the paired samples *t*-test showed a systematic error for those two parameters. 

Table 10 reports no agreement for stance percentage, swing percentage, and stride length (*p* > 0.05), while there is agreement for all the other variables. In particular, stance time shows a good correlation (ICC = 0.662), while swing time and cycle time show excellent agreement (ICC = 0.765 and ICC = 0.889) [25].

Table 11 shows the ICC for each parameter measured with Move4D and Xsens for the second trial. Two-way mixed effects, absolute agreement, and single-rater ICC have been computed for all parameters except stride length. For stride length a, two-way mixed effects, consistency, and single rater ICC have been evaluated since the previous results on the paired samples *t*-test on trial two showed a systematic error for this parameter. 

As before, Table 11 reports no agreement for stance percentage, swing percentage, and stride length (*p* > 0.05), while there is agreement for all the other variables. In particular, swing time shows a good correlation (ICC = 0.601), while stance time and cycle time show excellent agreement (ICC = 0.806 and ICC = 0.869) [25].

Table 12 shows the ICC for each parameter measured with Move4D and Xsens for the third trial. The ICCs for the third trial have been computed as the ICCs of the second trial.

Table 12 shows an excellent correlation for stance time (ICC = 0.760), swing time (0.788), and cycle time (0.897).

### 3.5. Kinematics

**Figure 4:**
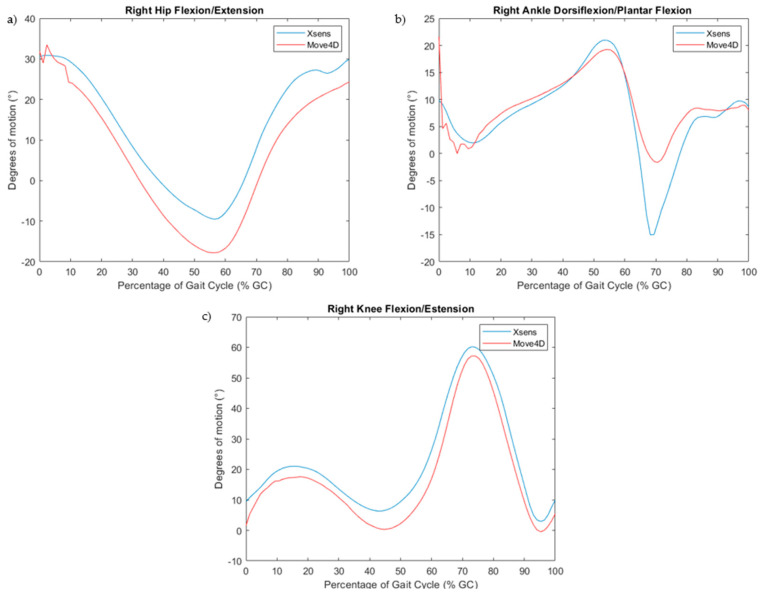
(**a**) Hip flexion (+)/extension (−); (**b**) knee flexion (+)/extension (−); (**c**) ankle dorsiflexion (+)/plantarflexion (−).

A kinematic analysis was performed on the first trial. Figure 4 shows examples of joint angles, particularly hip and knee flexion/extension and ankle dorsiflexion/plantar flexion, obtained with Move4D and Xsens for a subject.

Table 13 shows the mean, standard deviation, and 95% confidence interval of the range of motion of the hip, knee, and ankle on the sagittal plane for the two measurement systems for the healthy subjects. The higher difference, as observed, is regarding the ankle dorsiflexion/plantarflexion.

Table 14 reports the values of the range of motion of the hip, knee, and ankle in the sagittal plane obtained with Move4D and Xsens for the hemiplegic patient. If, for Move4D, we compare the mean of hip and knee flexion/extension of the hemiplegic patient with the 95% CI obtained for the healthy subjects, it is clear that the hemiplegic patient is outside the normal range. The same is true for the knee flexion/extension measured with Xsens.

Table 15 shows the RMSE between the two measurement systems in estimating the hip, knee, and ankle ranges of motion in the sagittal plane. The most significant difference is hip flexion/extension (RMSE 10.99°).

## 4. Discussion

In the present study, thirteen healthy subjects and one hemiplegic patient performed one complete gait cycle acquired simultaneously with two measurement systems, Move4D and Xsens. Xsens [26] is a wearable mocap system suitable for synchronization with Move4D. The systems have been synchronized to understand the degree of agreement needed to evaluate the spatiotemporal parameters of gait and the joint angles for the lower limb. The results obtained for the inter-trial variability show that Move4D can guarantee repeatability over time. This achievement represents an advantage as there is no need to repeat the measurement many times, making the system significantly less time-consuming. This result is primarily essential when patients with gait disorders are evaluated since fatigue can affect them after many trials, making the movement not natural anymore. Additionally, for Xsens, the results show no variability between trials. In this case, the system continues to be time-consuming because it involves positioning the inertial sensors over straps and calibrating each subject. Moreover, for the stride length, the variability between trials for Xsens is higher than the one for Move4D (Table 2). 

The most notable point is that the mean values of the spatiotemporal parameters for healthy subjects are within a physiological range, which agrees with the literature [5]. The mean stance and swing phases are split in 60% for stance and stance and 40% for swing. 

Additionally, the results obtained for the cycle time are comparable with the literature [27]. In some cases, observing Table 3, Table 4 and Table 5, the values of the cycle time are slightly higher (maximum is 1.26 s) than the reference (between 0.98 s and 1.07 s [27]). The causes for such results can be different. For example, it is known that the walking speed influences the gait parameters [28,29,30]. The two systems do not show high variability between subjects. However, in this case, the stride length variability for Xsens (SD = 0.13 m) is more than two times more significant than for Move4D (SD = 0.05 m).

According to the literature, a hemiplegic patient should show a shorter stride length, a longer stride duration, and a shorter swing duration [30,31]. The results for the hemiplegic patient obtained in this study (Table 6) are coherent with what was expected, both for Move4D and Xsens. The most important result is the ability of the Move4D system to distinguish between healthy and pathological subjects (Table 2 and Table 6).

Table 7, Table 8 and Table 9 show that, over the three trials, the stride length is the parameter that has a significant difference between the two measurement systems. The reasons for that result are different. First, since the difference occurs in all the trials, it can be related to the sensors’ attachment to the foot. The sensors have been placed over the shoes where friction phenomena with the sensor can occur. The other problem related to sensor attachment is that the shape of the shell containing the inertial measurement units can affect the reconstruction of the subjects’ mesh. It would be better to wear sensors as small as possible or not at all. Moreover, according to He et al. [12], inertial measurement unit noise, in general, affects the accuracy of stride length estimation. In the present study, the stride length measured with Xsens seems underestimated, according to the results obtained in the survey by Scataglini et al. [32], where Xsens has been compared with photoelectronic systems.

In addition, if we also consider that the subjects were healthy, we can then consider studies in the literature that use optoelectronic marker-based cameras (e.g., VICON, BTS, Qualisys) that assess the same age-specific spatiotemporal parameters of gait. Indeed, in the study of Pietraszewski et al. [33], seventeen students (22.0 ± 1.0 y) were captured using the gold standard optoelectronic marker-based systems for assessing gait analysis. They found that at the preferred walking speed of 1.36 ± 0.17 m/s, the stride length is 1.47 ± 0.13 m. This result aligns with our study using Move4D. However, in both systems, Xsens and Move4D, the cycle times (Table 4 and Table 5) were around 1.22–1.24 s.

Considering that the stride length is equal to the speed divided by the cycle time [34] and assuming a mean stride length of 1.11–1.13 m (Table 4 and Table 5), by using the Xsens, it appears a speed of 0.8–0.9 m/s. This result is very low for a healthy subject. It is more related to a pathological subject [35]. Considering a mean stride length of 1.40–1.46 m for Move4D (Table 4 and Table 5), we obtain approximately a speed of 1.2 m/s that aligns with the literature on healthy subjects [34]. 

Regarding reliability, Bland–Altman plots (Figure A1, Figure A2, Figure A3, Figure A4, Figure A5, Figure A6, Figure A7, Figure A8 and Figure A9) show agreement for all the spatiotemporal parameters obtained with Move4D and Xsens except for the stride length. 

Computing the ICC [36] as a measurement for inter-trial and inter-subject reliability is recommended. The ICC shows an excellent correlation [24] for the stance time, swing time, and cycle time estimation. The results for all three trials show a poor correlation between the measurements obtained for Move4D and Xsens of the stride length.

The results obtained for the values of hip, knee, and ankle range of motion (Figure 4, Table 13) align with what is expected in the physiology of healthy subjects [5]. 

For the hemiplegic patient, a lower hip and knee flexion/extension than normal are found, according to the literature [37]. Additionally, in this case, Move4D clearly distinguishes between healthy and pathological subjects (Table 14). Regarding the RMSE (Table 15) on evaluating the range of motion between Move4D and Xsens, the results are comparable with the study of Mavor et al. [38], where an IMU system is compared with an optical motion capture system to assess joint kinematics, and the values of RMSE obtained range between 0° and 10°. 

In our study, the highest values for the RMSE are found for the hip (RMSE = 10.99°) and ankle (RMSE = 10.25°). The reason could be that IMUs require attachment to the human body by positioning the sensors with Velcro straps, which can be unstable and cause movement artifacts [32]. Moreover, automatically estimating the IMUs sensor’s position from the Xsens integration of the acceleration could introduce a misalignment error [39]. Despite this problem, IMUs are now a portable solution for assessing gait analysis. Consequently, we compared them with 4D stereophotogrammetry for gait spatiotemporal parameters and joint kinematics. 

In this study, we first explore the use of 4D scanning technology [18,22], such as Move4D, for assessing spatiotemporal parameters of gait. Move4D allows for capturing the kinematic model [40], together with the dynamic full-body shape of the subject, to represent a personalized digital human model [41] of the subject during walking. However, it has some limitations: due to a limited time of acquisition (in this case three seconds) it is possible to capture only one or two gait cycles; it is not a portable solution; the processing and storage of the data is time consuming and they requires a big amount of space (from gigabyte to terabyte); and the subject needs to wear tight-fitting clothes for capture. 

Move4D has been compared with the full body inertial measurement system Xsens. 

Xsens has been demonstrated to be a valued portable instrument for gait assessment for long term monitoring [11,12]. Nevertheless, it requires skilled personnel to attach inertial measurement units on the body making the procedure time consuming and limiting the free natural movement of the end user. 

Move4D solves this issue ensuring a no time-consuming procedure because it does not involve the use of markers or sensors, making it particularly useful when patients with gait disorders need to be evaluated. Considering all the drawbacks of markers and sensors positioning in optical and inertial motion capture systems is a great advantage. Furthermore, Move4D allows for simultaneous information about the body shape, possibly combining gait analysis with body composition and musculoskeletal modeling. It also enables the evaluation of soft tissue deformation. 

Results achieved in this study will open new frontiers in gait analysis—4D scanning can be employed for gait analysis of pathological patients, where it is necessary to compare the alteration of gait patterns with the shape (e.g., obesity [42]).

Further improvements can be applied to the present work. First, it would be necessary to enlarge the number of subjects, especially for the patients. It would be interesting to have the same number of patients and healthy subjects and the same number of females and males. Furthermore, the analysis should not be limited to the lower part of the body but should also consider the upper part.

## 5. Conclusions

The present study compared a 3D temporal scanning system, Move4D, with an inertial motion capture system, Xsens, for gait assessment. The study compared the kinematic data (joint angles) and the main spatiotemporal parameters. To our knowledge, it is the first attempt to extract such parameters from a 4D scanner. The degree of agreement between the two measurement systems has been evaluated for each parameter and joint angle. Moreover, the results have also been compared with literature to demonstrate their physiological meaning.

The results showed that for both Move4D and Xsens, the values of the spatiotemporal parameters and the kinematic data are coherent with the literature; thus, the systems provide results that can be clinically interpreted. 

One of the present study’s most significant achievements is highlighting the ability of Move4D to distinguish between healthy and pathological patients.

A high degree of agreement between the two measurement systems has been found for the stance, swing, and cycle times. The kinematic analysis further proves these results. The slight differences in estimating the range of motion between Move4D and Xsens align with the literature.

In conclusion, the present work demonstrated that the 4D stereophotogrammetry system (Move4D) can estimate gait spatiotemporal parameters and kinematic data with reliability and accuracy comparable with Xsens. The results open new frontiers of using and empowering a new non-invasive markerless technology based on 3D temporal scanning for gait analysis.

## Figures and Tables

**Table 1:** Anthropometric characteristics of the study population.

	Mean	SD
Age (years)	23.30	1.44
Height (m)	1.72	0.06
Weight (kg)	70.44	7.37
BMI (kg/m^2^)	23.85	2.69

**Table 2:** Mean, standard deviation, and 95% CI of the measurements between three trials of 13 healthy subjects.

	MOVE4D			
	Mean	STD	95% CI	
			Upper	Lower
Stance time (s)	0.75	0.03	0.81	0.69
Swing time (s)	0.49	0.02	0.53	0.46
Stance perc. (%)	60.27	1.12	62.72	57.82
Swing perc. (%)	39.73	1.12	42.18	37.28
Cylce time (s)	1.24	0.03	1.31	1.17
Stride length (m)	1.43	0.07	1.58	1.28
	**Xsens**			
	**Mean**	**STD**	**95% CI**	
			Upper	Lower
Stance time (s)	0.75	0.03	0.82	0.69
Swing time (s)	0.50	0.02	0.53	0.46
Stance perc. (%)	60.26	1.05	62.56	57.97
Swing perc. (%)	39.74	1.05	42.03	37.44
Cylce time (s)	1.25	0.04	1.33	1.17
Stride length (m)	1.12	0.15	1.44	0.80

**Table 3:** Mean, standard deviation, and 95% CI of the measurements on the 13 healthy subjects (first trial). The values are reported both for Move4D and Xsens.

TRIAL1								
	MOVE4D				Xsens			
	Mean	STD	95% CI of the Mean		Mean	STD	95% CI of the Mean	
			Upper	Lower			Upper	Lower
Stance time (s)	0.74	0.06	0.78	0.71	0.77	0.07	0.81	0.72
Swing time (s)	0.49	0.03	0.51	0.47	0.5	0.03	0.52	0.48
Stance perc. (%)	60.15	1.97	61.34	58.96	60.69	1.89	61.83	59.55
Swing perc. (%)	39.85	1.97	41.04	38.66	39.31	1.89	40.45	38.17
Cylce time (s)	1.24	0.08	1.29	1.19	1.26	0.09	1.32	1.21
Stride length (m)	1.4	0.07	1.44	1.36	1.13	0.22	1.27	1.00

**Table 4:** Mean, standard deviation, and 95% CI of the measurements on the 13 healthy subjects (second trial). The values are reported both for Move4D and Xsens.

TRIAL2								
	MOVE4D				Xsens			
	Mean	STD	95% CI of the Mean		Mean	STD	95% CI of the Mean	
			Upper	Lower			Upper	Lower
Stance time (s)	0.74	0.06	0.78	0.70	0.74	0.08	0.79	0.69
Swing time (s)	0.49	0.03	0.51	0.47	0.49	0.03	0.51	0.48
Stance perc. (%)	59.96	1.66	60.96	58.96	60.05	1.87	61.18	58.92
Swing perc. (%)	40.04	1.66	41.04	39.04	39.95	1.87	41.08	38.82
Cylce time (s)	1.23	0.08	1.28	1.18	1.24	0.11	1.30	1.17
Stride length (m)	1.46	0.09	1.51	1.40	1.11	0.25	1.26	0.96

**Table 5:** Mean, standard deviation, and 95% CI of the measurements on the 13 healthy subjects (third trial). The values are reported both for Move4D and Xsens.

TRIAL3								
	MOVE4D				Xsens			
	Mean	STD	95% CI of the Mean		Mean	STD	95% CI of the Mean	
			Upper	Lower			Upper	Lower
Stance time (s)	0.74	0.05	0.77	0.71	0.73	0.07	0.77	0.69
Swing time (s)	0.49	0.03	0.50	0.47	0.49	0.03	0.51	0.47
Stance perc. (%)	60.43	1.15	61.12	59.74	59.68	2.24	61.03	58.32
Swing perc. (%)	39.57	1.15	40.26	38.88	40.32	2.24	41.68	38.97
Cylce time (s)	1.23	0.07	1.27	1.19	1.22	0.08	1.27	1.17
Stride length (m)	1.43	0.07	1.47	1.39	1.12	0.19	1.24	1.01

**Table 6:** Mean, standard deviation, and 95% CI of the spatiotemporal parameters between three trials for the hemiplegic patient. The values are reported both for Move4D and Xsens.

Hemiplegic Patient				
	MOVE4D			
	Mean	STD	95% CI of the Mean	
			Upper	Lower
Stance time (s)	0.99	0.12	1.24	0.73
Swing time (s)	0.51	0.05	0.61	0.39
Stance perc. (%)	66.14	0.46	67.14	65.14
Swing perc. (%)	33.86	0.46	34.85	32.85
Cylce time (s)	1.49	0.17	1.85	1.12
Stride length (m)	0.94	0.08	1.09	0.77
	**Xsens**			
	**Mean**	**STD**	**95% CI of the Mean**	
			Upper	Lower
Stance time (s)	0.90	0.15	1.21	0.58
Swing time (s)	0.53	0.05	0.64	0.42
Stance perc. (%)	62.64	2.39	67.85	57.43
Swing perc. (%)	37.35	2.39	42.56	32.15
Cylce time (s)	1.43	0.19	1.84	1.02
Stride length (m)	0.80	0.16	1.16	0.44

**Table 7:** Paired sample *t*-test between the measurements of the spatiotemporal parameters obtained with Move4D and Xsens for the first trial.

Paired Differences							
	Mean	SD	Std. Error Mean	95% CI		t	Significance
				Lower	Upper		Two-Sided *p*
Stance time (s)	−0.023	0.054	0.015	−0.056	0.01	−1.534	0.151
Swing time (s)	−0.004	0.022	0.006	−0.017	0.009	−0.640	0.534
Stance perc. (%)	−0.535	2.530	0.702	−2.064	0.994	−0.763	0.460
Swing perc. (%)	0.535	2.530	0.702	−0.994	2.064	0.763	0.460
Cycle time (s)	−0.027	0.041	0.011	−0.052	−0.002	−2.360	0.036
Stride length (m)	0.272	0.23	0.064	0.133	0.411	4.273	0.001

**Table 8:** Paired sample *t*-test between the measurements of the spatiotemporal parameters obtained with Move4D and Xsens for the second trial.

Paired Differences							
	Mean	SD	Std. Error Mean	95% CI		t	Significance
				Lower	Upper		Two-Sided *p*
Stance time (s)	−0.005	0.047	0.013	−0.034	0.023	−0.391	0.703
Swing time (s)	0.000	0.027	0.008	−0.016	0.016	0.000	1.000
Stance perc. (%)	−0.088	2.097	0.582	−1.355	1.179	−0.152	0.882
Swing perc. (%)	0.088	2.097	0.582	−1.179	1.355	0.152	0.882
Cycle time (s)	−0.005	0.051	0.014	−0.036	0.025	−0.365	0.721
Stride length (m)	0.343	0.283	0.079	0.171	0.514	4.356	<0.001

**Table 9:** Paired sample *t*-test between the measurements of spatiotemporal parameters obtained with Move4D and Xsens for the third trial.

Paired Differences							
	Mean	SD	Std. Error Mean	95% CI		t	Significance
				Lower	Upper		Two-Sided *p*
Stance time (s)	0.015	0.039	0.011	−0.008	0.039	1.409	0.184
Swing time (s)	−0.004	0.019	0.005	−0.016	0.008	−0.714	0.489
Stance perc. (%)	0.754	1.905	0.528	−0.397	1.905	1.427	0.179
Swing perc. (%)	−0.754	1.905	0.528	−1.905	0.397	−1.427	0.179
Cycle time (s)	0.012	0.034	0.010	−0.009	0.032	1.214	0.248
Stride length (m)	0.308	0.180	0.050	0.200	0.417	6.181	<0.001

**Table 10:** Intraclass correlation coefficient (ICC) for each parameter was measured with MOVE4D and Xsens for the first trial. Two-way mixed effects, absolute agreement, and single-rater ICC have been computed for all parameters except cycle time and stride length. In these two cases, two-way mixed effects, consistency, and single rater ICC have been evaluated, as the previous results on the paired samples *t*-test showed a systematic error for those two parameters.

Intraclass Correlation Coefficient TRIAL1				
	Intraclass Correlation	95% Confidence Interval		Significance
		Lower Bound	Upper Bound	
Stance time (s)	0.662	0.230	0.880	0.004
Swing time (s)	0.765	0.400	0.920	<0.001
Stance perc. (%)	0.143	−0.440	0.630	0.318
Swing perc. (%)	0.143	−0.440	0.630	0.318
Cycle time (s)	0.889	0.677	0.965	<0.001
Stride length (m)	0.010	−0.530	0.540	0.491

**Table 11:** The intra-class correlation coefficient for spatiotemporal parameters obtained with Move4D and Xsens for the second trial.

Interclass Correlation Coefficient TRIAL2				
	Intraclass Correlation	95% Confidence Interval		Significance
		Lower Bound	Upper Bound	
Stance time (s)	0.806	0.476	0.937	<0.001
Swing time (s)	0.601	0.077	0.860	0.015
Stance perc. (%)	0.311	−0.311	0.731	0.153
Swing perc. (%)	0.311	−0.311	0.731	0.153
Cycle time (s)	0.869	0.626	0.958	<0.001
Stride length (m)	−0.150	−0.632	0.416	0.696

**Table 12:** Intra-class correlation coefficient for spatiotemporal parameters obtained with Move4D and Xsens for the third trial.

Intraclass Correlation Coefficient TRIAL3				
	Intraclass Correlation	95% Confidence Interval		Significance
		Lower Bound	Upper Bound	
Stance time (s)	0.760	0.400	0.919	<0.001
Swing time (s)	0.788	0.448	0.93	<0.001
Stance perc. (%)	0.407	−0.110	0.765	0.065
Swing perc. (%)	0.407	−0.110	0.765	0.065
Cycle time (s)	0.897	0.707	0.967	<0.001
Stride length (m)	0.196	−0.375	0.660	0.251

**Table 13:** Mean, standard deviation, and 95% confidence interval of the hip, knee, and ankle range of motion on the sagittal plane for the two measurement systems for the first trial of healthy subjects.

	MOVE4D				Xsens			
	Mean	STD	95% CI		Mean	STD	95% CI	
			Upper	Lower			Upper	Lower
Hip flexion/extension (°)	44.87	2.54	50.41	39.33	41.07	2.96	47.52	34.63
Knee flexion/extension (°)	58.75	4.14	67.78	49.72	61.06	3.76	69.24	52.88
Ankle dorsiflexion/plantarflexion (°)	24.13	7.67	40.84	7.42	35.49	6.23	49.05	21.92

**Table 14:** Values of the range of motion of the hip, knee, and ankle in the sagittal plane obtained with Move4D and Xsens for the hemiplegic patient on the first trial.

	Move4D	Xsens
Hip flexion/extension (°)	31.41	42.25
Knee flexion/extension (°)	29.71	27.39
Ankle dorsiflexion/plantarflexion (°)	39.24	41.19

**Table 15:** RMSE for estimating hip, knee, and ankle range of motion between Move4D and Xsens.

	Hip Flexion/Extension (°)	Knee Flexion/Extension (°)	Ankle Dorsiflexion/Plantarflexion (°)
RMSE	10.99	5.07	10.25

## Data Availability

Data are contained within the article.
